# A (fire)cloud-based DNA methylation data preprocessing and quality control platform

**DOI:** 10.1186/s12859-019-2750-4

**Published:** 2019-03-29

**Authors:** Divy Kangeyan, Andrew Dunford, Sowmya Iyer, Chip Stewart, Megan Hanna, Gad Getz, Martin J. Aryee

**Affiliations:** 1 Department of Biostatistics, Harvard T. H. Chan School of Public Health, Boston, MA USA; 2 Broad Institute of MIT & Harvard, Cambridge, MA USA; 3 Department of Pathology, Massachusetts General Hospital, Boston, MA USA; 4 Department of Pathology, Harvard Medical School, Boston, MA USA; 5 Cancer Center, Massachusetts General Hospital, Boston, MA USA

**Keywords:** DNA methylation, Cloud computing, Bioinformatics workflows, Quality control analysis

## Abstract

**Background:**

Bisulfite sequencing allows base-pair resolution profiling of DNA methylation and has recently been adapted for use in single-cells. Analyzing these data, including making comparisons with existing data, remains challenging due to the scale of the data and differences in preprocessing methods between published datasets.

**Results:**

We present a set of preprocessing pipelines for bisulfite sequencing DNA methylation data that include a new R/Bioconductor package, *scmeth*, for a series of efficient QC analyses of large datasets. The pipelines go from raw data to CpG-level methylation estimates and can be run, with identical results, either on a single computer, in an HPC cluster or on Google Cloud Compute resources. These pipelines are designed to allow users to 1) ensure reproducibility of analyses, 2) achieve scalability to large whole genome datasets with 100 GB+ of raw data per sample and to single-cell datasets with thousands of cells, 3) enable integration and comparison between user-provided data and publicly available data, as all samples can be processed through the same pipeline, and 4) access to best-practice analysis pipelines. Pipelines are provided for whole genome bisulfite sequencing (WGBS), reduced representation bisulfite sequencing (RRBS) and hybrid selection (capture) bisulfite sequencing (HSBS).

**Conclusions:**

The workflows produce data quality metrics, visualization tracks, and aggregated output for further downstream analysis. Optional use of cloud computing resources facilitates analysis of large datasets, and integration with existing methylome profiles. The workflow design principles are applicable to other genomic data types.

## Background

DNA methylation is an essential component of the epigenetic machinery that regulates gene expression. It involves a chemical modification whereby a methyl group is added to Cytosine bases [1]. DNA methylation is highly dynamic during development and aberrations in the mark are associated with a range of diseases including cancer, autoimmune and neurodegenerative disorders [2–5].

The gold-standard assays for DNA methylation are based on bisulfite sequencing, where unmethylated cytosines (C) are selectively and efficiently converted to thymines (T) allowing base-pair resolution methylation state to be read out by standard high-throughput sequencing [6]. Bisulfite sequencing can be applied to a whole genome library (WGBS) [7], or in targeted variants that include Reduced Representation Bisulfite Sequencing (RRBS) [8] that enriches for regions of high CpG density, and Hybrid Selection Bisulfite Sequencing (HSBS) [9] that uses capture probes to target a specific set of genomic regions of interest.

Preprocessing and quality control typically comprise the most computationally intensive portion of bisulfite sequencing data analysis, due to the large size of raw datasets which may contain > 100 GB of data for deeply sequenced individual samples, or thousands of cells in single-cell projects [10]. Here we present a set of preprocessing tools for bisulfite sequencing data that facilitate analyses of such datasets, by simplifying, and making more accessible, the use of large computational compute clusters. We also introduce a new R/Bioconductor package, *scmeth*, that is optimized for QC analysis of large datasets. The pipelines can be run locally or on cloud computing infrastructure, providing practically unlimited scalability without requiring local compute resources. The cloud implementation, in particular, is accessible through a web browser interface and lends itself to both researchers who have technical expertise and to users with limited bioinformatics analysis experience.

## Implementation

The pipelines are designed to go from raw sequencing data to CpG-level methylation estimates. The workflows first perform read alignment and methylation calling in parallel across samples, followed by an aggregation and quality control analysis step. The workflows are implemented in the WDL workflow description language [11, 12] and use software packaged into Docker [13] containers. WDL files are typically structured to contain a workflow consisting of one or more tasks. Both workflows and tasks can specify input parameters such as raw read file names, and runtime parameters such as the amount of CPU and memory resources required for processing and the specific docker image to use. Docker containers are lightweight virtual machines that encapsulate the entire software environment required by the pipeline tools, including their dependencies. In addition to the option of running the WDL workflows locally on a single computer or on an HPC (High-Performance Computing) cluster using job management systems such as LSF (Load Sharing Facility) or SLURM (Simple Linux Utility for Resource Management), we also provide an implementation that is available through the Google Cloud-based FireCloud platform [14, 15]. FireCloud is accessible through a web-browser and allows execution of WDL-based workflows on cloud compute resources with scalability that is unlimited for most practical use cases. The *scmeth* package used for QC analysis is part of the R/Bioconductor project.

## Results

**Fig. 1:**
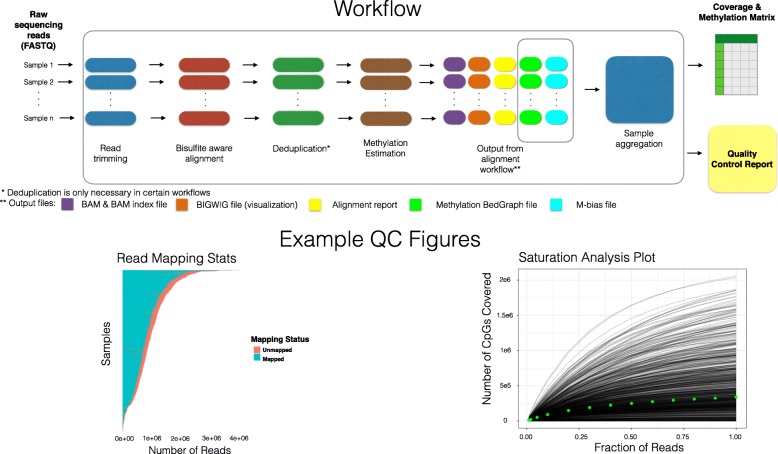
Overview of methylation analysis workflow. Raw read (FASTQ) files and are first processed through a per-sample alignment and pre-processing step, followed by an aggregation step that combines data from all samples into a matrix format and generates a QC report

The methylation workflows follow a two-step pattern, with a parallelized per-sample preprocessing step followed by an aggregation and QC step that integrates data across the dataset. Following initial preprocessing with the pipeline default bisulfite-aware aligner Bismark [16], the following outputs are generated for each input sample: (i) BAM and BAM index files; (ii) a per-CpG coverage file with unmethylated and methylated read counts; (iii) a bigwig file for visualization, and (iv) a set of quality assessment metrics such as fraction of aligned reads, bisulfite conversion rate and methylation value distributions. The aggregation step then prepares the individual sample outputs for downstream analysis by combining them into coverage and methylation matrices, available either as plain text or as an R/Bioconductor *bsseq* [17] object that is also annotated with metrics including the number of reads, number of covered CpGs and bisulfite conversion rate (Fig. 1).

In addition to preprocessed methylation data, comprehensive HTML and plain text quality reports are also generated using tools implemented in the *scmeth* Bioconductor package [18]. The QC report can be used to identify low quality batches or samples, and provides metrics, including number of reads, total CpG coverage, bisulfite conversion rate, methylation distribution, genomic feature coverage (e.g. promoters, enhancers), a downsampling saturation curve and methylation distributions (Table 1). In order to scale to large sample sizes as is common in single-cell analysis, an on-disk representation of the methylation and coverage matrices as implemented in the *bsseq* [17] package is used by default. In order to improve QC analysis run time for large datasets, *scmeth* provides an option to subsample while calculating metrics. We find that estimates based on using as few as one million of the ~ 28 million CpGs in the human genome are unbiased and stable.

**Table 1:** Quality control metrics

QC Metric	Information gained from this metric
Read metrics	Number of mapped and unmapped reads
CpG Coverage	Number of CpGs observed with a minimum coverage threshold
M-bias	Average methylation by position across reads. Deviation from uniformity typically indicates a problem with library construction or data preprocessing.
Downsampling saturation curve	CpG coverage as a function of number of reads. A rising curve indicates that we would expect to observe additional CpGs from deeper sequencing of a library
CpG discretization	Useful in single-cell analysis, this represents the fraction of CpGs with non-binary methylation status
Feature level coverage	The fraction of key genomic features (e.g. promoters, CpG Islands), covered with at least 1 CpG.
Bisulfite conversion rate	The proportion of non CpG context C’s that were converted to T. This should be close to 100% in most mammalian tissues.
CpG density distribution	The CpG density distribution around observed CpGs is typically similar across samples indicating coverage of similar genomic regions.
Methylation distribution	Unexpected sample-to-sample deviations in the distribution of methylation values can indicate potential technical artifacts

We used 1000 single-cell RRBS samples with a median of 872,223 reads (range of 5437 to 4,165,149) to estimate the run time and cost for the workflows. For example, processing the full set of 1000 samples using default options took 62 h and accrued $66 of Google Cloud charges (Table 2).

**Table 2:** Run time and cost estimates

Sample size	Per-sample preprocessing (Hours/$)	Aggregation and QC (Hours/$)	Total (Hours/$)
10	0.98 ($0.93)	0.97 ($0.28)	1.95 ($1.21)
100	1.47 ($8.99)	6.00 ($0.86)	7.47 ($9.85)
1000	4.48 ($52.48)	58.01 ($13.74)	62.49 ($66.22)

Time and cost to conduct various steps of the workflow with different sample sizes. Estimates were obtained when the workflows were run on the default n1-highmem-4 compute nodes (26 GB RAM with 4 CPUs) in FireCloud. Note that these example times and costs will decrease considerably as workflows are improved and compute resources become cheaper

### TCGA data analysis

We have preprocessed and made available 47 WGBS samples available from TCGA. These samples were sequenced with a median of 361,777,141 reads (range of 289,476,432 to 955,974,014). We confirmed a high concordance in methylation estimates with the available BEDgraph files from the NCI Genomic Data Commons (GDC), with a correlation of 0.99 when considering CpGs with a minimum read coverage 10. The raw (FASTQ) data, processed data and workflows are made available in a FireCloud workspace (See https://github.com/aryeelab/dna-methylation-tools/blob/master/README.md#tcga-data). We have also made the processed data available via tcgaWGBSData.hg19, an experiment data package in Bioconductor.

The workflows are pre-configured with the quantity of compute resources (e.g. memory and number of CPU cores) to request from either an HPC system (e.g. LSF) or the cloud environment for each analysis step, but these can be altered by the user if a different tradeoff between run time and cost is desired [15].

### Discussion

To guarantee reproducible analyses, we take advantage of two components: First, we use a workflow description language, WDL, that can be executed without modifications on systems ranging from a laptop, to an HPC cluster, to cloud compute resources. This flexibility is provided by the workflow engine, Cromwell [15], which has various “back-ends” allowing it to execute workflow tasks on the various platforms. Second, we use Docker containers, lightweight virtual machines, that package the full software environment required by the pipeline tools. These two components together ensure that identical results are produced across different platforms and across multiple runs of the pipelines.

Scalability is achieved through parallelization across samples. For users with an HPC cluster that supports Docker containers, this parallelization is accessible locally. Alternatively, any user can take advantage of the FireCloud platform that uses the Google Compute Engine as the computing platform. The Google billing model charges per minute per machine, which enables all per-sample preprocessing to be performed within a near-fixed total time, regardless of the number of samples, as all samples can be processed in parallel. There are no additional charges for using the FireCloud platform itself although the user will accrue compute and storage costs billed by Google for resources used in workflow execution.

When analyzing a new dataset, it is often useful to compare the new samples to public data, either from individual published studies or large consortia like TCGA [19] and TARGET [20]. These data are often not directly comparable, however, due to differences in preprocessing and other upstream analysis. Applying a uniform processing pipeline is, on the other hand, challenging due to the size of the datasets (e.g. TCGA) making them difficult to download and process. As FireCloud already hosts raw TCGA data, an alternative is to take advantage of our DNA methylation workflow to process both TCGA and the user’s own data in a uniform manner on this platform. The preprocessed data, which is much smaller than the raw sequencing data, can then either be further analyzed using cloud resources, or downloaded for local downstream analysis.

## Conclusion

We have developed a set of preprocessing and quality assessment pipelines for Bisulfite sequencing-based DNA Methylation analysis. By leveraging Docker containers and a workflow language that can be executed both locally and in the cloud, the pipelines produce reproducible output across different platforms and user environments. This also has the benefit of facilitating comparisons across datasets such as between local user data and data from public repositories (e.g. TCGA) as identical preprocessing can be guaranteed. We have also introduced the *scmeth* R/Bioconductor package that implements QC functions optimized for large methylation datasets, such as those common in single-cell analyses. We take advantage of the pipelines’ portability by providing an implementation in the Google Cloud-based FireCloud platform, which enables any user the ability to scale to very large datasets without local compute capacity restraints. We believe that these tools will be useful as the scale of DNA methylation datasets grow, and that they will serve as a template for tools for other types of large genomic data.

## Availability and requirements

Project Documentation: http://aryee.mgh.harvard.edu/dna-methylation-tools/

Firecloud workspace: https://portal.firecloud.org/#workspaces/aryee-lab/dna-methylation (Users need to create a free account).

Operating System(s): Platform independent.

Programming Language: WDL, R.

License: MIT.

Any restrictions to use by non-academics: None.

Documentation for this pipeline and all the workflows can be accessed at http://aryee.mgh.harvard.edu/dna-methylation-tools/. *scmeth* is available through the Bioconductor project (https://www.bioconductor.org/packages/release/bioc/html/scmeth.html).

## Abbreviations

HSBS: Hybrid Selection Bisulfite Sequencing
QC: Quality Control
RRBS: Reduced Representation Bisulfite Sequencing
TARGET: Therapeutically Applicable Research to Generate Effective Treatments
TCGA: The Cancer Genome Atlas
WGBS: Whole Genome Bisulfite Sequencing

## References

[1] Suzuki MM, Bird A (2008). DNA methylation landscapes: provocative insights from epigenomics. Nat Rev Genet.

[2] Okano, Masaki, et al. "DNA methyltransferases Dnmt3a and Dnmt3b are essential for de novo methylation and mammalian development." Cell 99.3 (1999): 247–257. 10.1016/s0092-8674(00)81656-6 10555141

[3] Messerschmidt DM, Knowles BB, Solter D (2014). DNA methylation dynamics during epigenetic reprogramming in the germline and preimplantation embryos. Genes Dev.

[4] Baylin SB, Jones PA (2011). A decade of exploring the cancer epigenome—biological and translational implications. Nat Rev Cancer.

[5] Al-Mahdawi S, Virmouni SA, Pook MA (2016). DNA methylation in neurodegenerative diseases. Epigenetic biomarkers and diagnostics.

[6] Frommer M, McDonald LE, Millar DS (1992). A genomic sequencing protocol that yields a positive display of 5-methylcytosine residues in individual DNA strands. Proc Natl Acad Sci U S A.

[7] Ziller MJ (2015). Coverage recommendations for methylation analysis by whole-genome bisulfite sequencing. Nat Methods.

[8] Meissner A (2005). Reduced representation bisulfite sequencing for comparative high-resolution DNA methylation analysis. Nucleic Acids Res.

[9] Ziller MJ (2016). Targeted bisulfite sequencing of the dynamic DNA methylome. Epigenetics Chromatin.

[10] Smallwood SA (2014). Single-cell genome-wide bisulfite sequencing for assessing epigenetic heterogeneity. Nat Methods.

[11] Voss K, Gentry J, Van der Auwera G (2017). Full-stack genomics pipelining with GATK4 + WDL + Cromwell [version 1; not peer reviewed]. F1000Research.

[12] WDL homepage. https://software.broadinstitute.org/wdl/. Accessed 29 Aug 2018.

[13] Docker homepage. https://www.docker.com/. Accessed 29 Aug 2018.

[14] FireCloud homepage. www.firecloud.org. Accessed 29 Aug 2018.

[15] Birger C, et al. FireCloud, a scalable cloud-based platform for collaborative genome analysis: strategies for reducing and controlling costs. bioRxiv. 2017:209494.

[16] Krueger F, Andrews SR (2011). Bismark: a flexible aligner and methylation caller for bisulfite-Seq applications. bioinformatics.

[17] Hansen KD, Langmead B, Irizarry RA (2012). BSmooth: from whole genome bisulfite sequencing reads to differentially methylated regions. Genome Biol.

[18] scmeth bioconductor page. https://www.bioconductor.org/packages/3.7/bioc/html/scmeth.html. Accessed 29 Aug 2018.

[19] Weinstein JN (2013). The cancer genome atlas pan-cancer analysis project. Nat Genet.

[20] National Cancer Institue, Office of Cancer Genomics. TARGET, Therapeutically Applicable Research To Generate Effective Treatments. 2013. https://www.cancer.gov/research/progress/discovery/target-initiative.

